# Acute glycogenic hepatopathy in pregnancy: a case report and literature review

**DOI:** 10.1515/crpm-2021-0065

**Published:** 2022-06-04

**Authors:** Omar Abuzeid, Mia Heiligenstein, Lama Noureddine, Cassandra Heiselman, James Bernasko

**Affiliations:** Division of Maternal Fetal Medicine, Department of Obstetrics and Gynecology and Reproductive Medicine, Renaissance School of Medicine at Stony Brook University, Stony Brook, NY, USA

**Keywords:** acute, glycogenic hepatopathy, type 1 diabetes mellitus

## Abstract

**Objectives:**

Acute glycogenic hepatopathy (AGH) is a rare complication of poorly controlled diabetes mellitus. This is the first report in the English literature describing accurate diagnosis and management of AGH during pregnancy.

**Case presentation:**

A 46 year-old gravida 4 para 2 presented at 30 weeks gestation with uncontrolled diabetes, ketoacidosis, and severe hypertension. Euglycemia and normotension were achieved within 24 h of admission but serum transaminase levels which had been normal on admission increased to a very high level over several days, and then resolved spontaneously.

**Conclusions:**

AGH may occur during pregnancy and should be considered in the context of chronic poorly controlled overt diabetes, rapid normalization of maternal blood glucose levels following high dose insulin therapy, and unexplained new-onset serum transaminase levels elevation. Accurate diagnosis is important because the correct treatment is conservative management, not delivery.

## Introduction

Pierre Mauriac in 1930 [1] reported a rare complication of pediatric type 1 diabetes characterized by growth failure, delayed puberty, Cushingnoid appearance, extreme liver enlargement due to glycogen deposition, abnormal liver enzymes and dyslipidemia [1]. Although the full syndrome as originally described is very rare, hepatic glycogen accumulation (aka glycogenic hepatopathy (GH) and hepatic glycogenosis) has recently been described more frequently recently including in type 2 diabetes [2]. The pathogenesis of this condition is an acute, severe, reversible intracytoplasmic accumulation of glycogen within hepatocytes resulting in hepatomegaly and transient elevation of liver function tests triggered by high dose insulin therapy and/or relatively rapid correction of chronic severe hyperglycemia [3]. Glucose diffuses into the liver via GLUT transporters leading to activation of phosphorylase and glucokinase which leads to formation of glycogen [4]. The reason for this rapid, excessive glycogen deposition is unclear, especially because the overwhelming majority of diabetic people never experience it. The condition is likely significantly underreported because it is not widely known.

Clinical presentation may include nausea, vomiting, right upper quadrant pain and hepatomegaly, severe hyperglycemia, diabetic ketoacidosis, and other signs of chronic poorly controlled diabetes, such as polyuria, polyphagia, and polydipsia [4]. Acute progressive liver dysfunction may result in jaundice, abdominal pain, and even ascites [5]. Risk factors to develop this disease include female gender, anorexia, extreme doses of corticosteroids, dumping syndrome following gastric bypass and poorly controlled diabetes mellitus [6]. Liver disease is common in people with chronic poorly controlled diabetes, with incidence varying between 17 and 100% [7]. Acute glycogenic hepatopathy (AGH) is usually clinically indistinguishable from non-alcoholic fatty liver disease (NAFLD) but it is important to be aware that the former does not cause liver fibrosis whereas the latter does [7]. The alanine aminotransferase (ALT) and alanine aminotransferase (AST) elevations in AGH invariably resolve completely within a few weeks.

The purpose of this case report is to describe the prenatal diagnosis and successful management of a pregnant patient with clinical and laboratory manifestations of AGH and hopefully increase awareness of this condition among healthcare practitioners.

### Case presentation

A 46 year-old gravida 4 para 2 presented to L&D at 30 weeks gestation complaining of one week of blurred vision, persistent headache and excessive urination. She had failed to follow up on a glucose challenge test (GCT) result of 222 mg/dL at 15 weeks gestation. A pregnancy 8 years earlier was complicated by diet treated gestational diabetes mellitus (GDM) and culminated in vaginal delivery after labor induction at 33 weeks for preeclampsia with severe features and postpartum Intensive Care Unit (ICU) admission for blood pressure control. She did not test for diabetes postpartum or at any time thereafter. She also had mild asthma since childhood. Her admission body mass index (BMI) was 36 kg/m^2^ and blood pressure (BP) was 185/90 mmHg. Preeclampsia with severe features was diagnosed, intravenous Hydralazine 5 mg and Labetalol 300 mg orally were given immediately, intravenous magnesium sulfate infusion was started, and Betamethasone 12 mg IM was given in anticipation of preterm delivery.

Pertinent admission results were HbA_1c_ – 12.5%, serum glucose – 626 mg/dL, anion gap – 31, serum bicarbonate – 12 mmol/L, beta-hydroxybutyrate – 7.9 mmol/L, serum creatinine – 1.21 mg/dL, ALT – 24 IU/L, AST – 22 IU/L, and negative urine toxicology. She refused ABG. Liver function tests, platelet count, lactate dehydrogenase (LDH), and urine protein were normal and anti-islet cell antibodies were negative. Diabetic ketoacidosis (DKA) was diagnosed, and an insulin drip, fluid infusion and electrolyte repletion were started.

The second dose of betamethasone was withheld because of hyperglycemia detected on admission. DKA resolved within 24 h of admission. NPH insulin was initiated after DKA resolved but was discontinued 24 h later because she developed a generalized allergic-type rash that was suspected to be drug-induced. Insulin glargine and Insulin lispro were started for basal-bolus therapy and adjusted according to fasting and postprandial blood glucose measurements to maintain blood glucose levels between 70 and 140 mg/dL. Diabetes self-management and nutrition education were provided. Labetalol, which had been initiated on admission at 300 mg BID, was discontinued approximately 36 h later due to persistent maternal mild hypotension. Antihypertensive therapy was never required for the pregnancy duration and postpartum. Maternal echocardiogram was normal. Ophthalmology and podiatry evaluations were unremarkable. The blurred vision (suspected to be due to hyperglycemia-induced intra-lenticular sorbitol accumulation) improved after hyperglycemia resolved, as expected. Fetal assessment was normal throughout the hospitalization.

**Figure 1:**
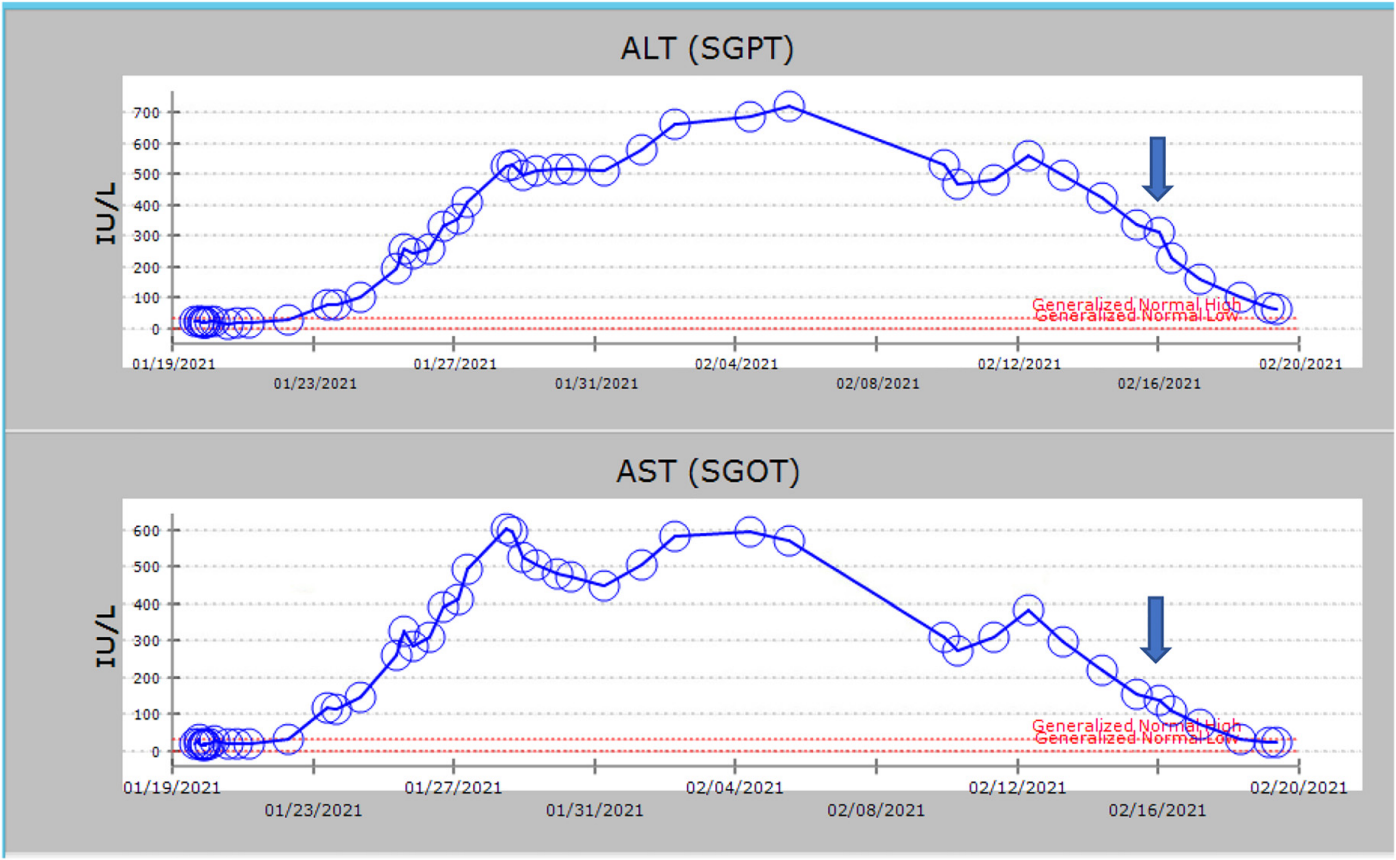
Graph illustrating liver transaminase trends during hospital course. Arrow indicates delivery date.

ALT and AST levels were normal on admission but began to rise two days after DKA resolved (Figure 1). Amylase and lipase levels were found to be slightly elevated on hospital day 4 (107 IU/L and 384 IU/L, respectively) but normalized over the next week. She denied abdominal pain, nausea or vomiting throughout the hospitalization and tolerated a full diet; therefore, acute pancreatitis was considered very unlikely. An extensive gastroenterology evaluation (hepatitis A IgM, hepatitis B sAg, hepatitis B cAb total, hepatitis E, HCV PCR, antinuclear antibody, anti-neutrophil cytoplasmic antibody, autoimmune hepatitis IgG, anti-mitochondrial antibodies, alpha-1 antitrypsin, urinalysis, urine toxicology screen, thyroid function test and anti-thyroid antibodies, anti-tissue transglutaminase antibody, anti-endomysial antibodies, ferritin, total iron-binding capacity (TIBC), and iron, serum ceruloplasmin level) was normal.

**Figure 2:**
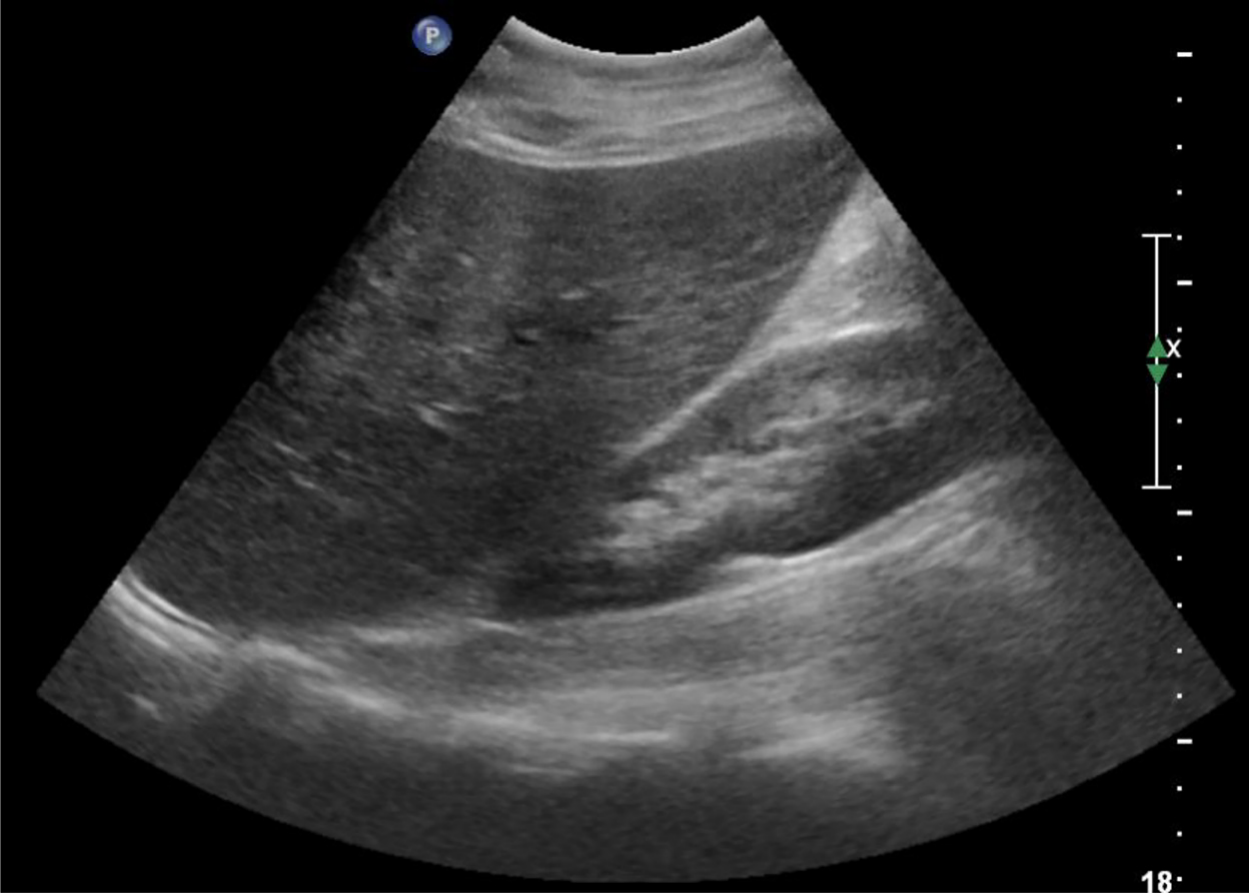
Right upper quadrant sonogram showing hepatomegaly.

Right upper quadrant ultrasound revealed moderate hepatomegaly (liver length of 18.7 cm) and increased echogenicity, but no cholelithiasis, acute cholecystitis or ductal dilatation (Figure 2). She declined magnetic resonance cholangiopancreatography (MRCP) and diagnostic liver biopsy. She first developed generalized itching twelve days after admission and serum bile acid level was 20 mg/dL. Ursodiol was started for presumed developing intrahepatic cholestasis of pregnancy. She left the hospital against medical advice on hospital day fourteen despite persistent itching and still elevated ALT and AST. She followed up outpatient for daily ALT/AST measurements, three times per week biophysical profiles (BPP), and weekly high risk obstetric clinic visits.

She agreed to return to the hospital at 33 weeks gestation and remain till delivery because of continued itching and persistently elevated ALT and AST despite Ursodiol 1,200 mg daily. Serum bile acids on this admission were 62 mmol/L. Readmission systolic BP ranged from 125 to 146 mmHg and diastolic BP ranged from 61 to 76 mmHg with +1 proteinuria. Preeclampsia with severe features was suspected initially, and a rescue course of betamethasone was administered. Intravenous magnesium infusion was started for seizure prophylaxis but was discontinued after twenty-four hours because her blood pressure remained normal. Her outpatient insulin regimen of insulin glargine 50 units qhs and insulin lispro 5 units before meals continued in hospital. Serum ALT and AST level peaked approximately fourteen days after she first presented in DKA and began to decrease spontaneously (Figure 1). Labor was induced at 34 weeks (ten days after the ALT, AST started decreasing) because of patient’s discomfort from persistent itching.

A male infant was delivered by emergency cesarean following a prolonged fetal bradycardia in the second stage of labor. APGAR scores were 3 and 8 at one and 5 min, respectively. The neonate was intubated for the first day of life because of poor respiratory effort, however he did well thereafter and did not experience hypoglycemia. Patient’s post-operative course was uncomplicated. The ALT and AST continued to trend down till discharge (Figure 1). Postoperative glucose control was excellent; therefore, insulin was replaced with metformin 1,000 mg BID. Patient consented to have her case reported in a medical journal.

## Discussion

This is the first report in the English literature that the authors are aware of that describes the prenatal diagnosis and successful management of AGH. Consideration of AGH is especially important in obstetrics because the common differential diagnoses of apparently unexplained serum transaminase elevation during pregnancy (specifically HELLP syndrome/pre-eclampsia with severe features, intrahepatic cholestasis of pregnancy, and acute fatty liver of pregnancy) often prompt iatrogenic delivery, even preterm. Cholestasis of any cause may result in generalized itching; therefore, obstetricians should not assume that all cholestasis that is encountered during pregnancy can only be ‘cholestasis of pregnancy’ (i.e. hormone-induced) which should strictly speaking be considered ‘a diagnosis of exclusion’. This patient’s ALT and AST began to decrease twelve days before she delivered which is strong evidence against any pregnancy-induced cause. In fact, pregnancy-induced causes were repeatedly proposed in this patient but were consistently not borne out by her clinical course. This patient’s presentation was complex: Advanced maternal age, high BMI, delayed treatment of early onset ‘gestational diabetes’, likely chronic hyperglycemia, diabetic ketoacidosis, transient severe hypertension, and AGH following institution of high dose insulin therapy and rapid correction of prolonged, severe hyperglycemia. There is no diagnostic serologic test for AGH and diagnosis is traditionally based on liver biopsy [8]; however, Khoury et al. (2018) [4] proposed that “GH may be diagnosed conservatively, based on corroborating medical history, physical examination, laboratory tests, imaging studies and response to treatment, even without liver biopsy” [4]. The negative extensive work-up is typical and further strengthens the case for AGH in this patient.

In conclusion, obstetricians should be aware of and consider AGH in the appropriate clinical situations, particularly because the correct treatment is not iatrogenic delivery but rather insulin therapy, adequate blood glucose control and standard fetal surveillance until spontaneous resolution of elevated transaminases.

### Take home message

AGH is rare during pregnancy. Acute glycogenic hepatopathy, like other diseases seen more frequently during pregnancy, for example, preeclampsia with severe features, causes elevated serum transaminases. Expectant management of acute glycogenic hepatopathy during pregnancy may avoid iatrogenic preterm birth.
